# mTORC1 as the main gateway to autophagy

**DOI:** 10.1042/EBC20170027

**Published:** 2017-12-12

**Authors:** Yoana Rabanal-Ruiz, Elsje G. Otten, Viktor I. Korolchuk

**Affiliations:** Institute for Cell and Molecular Biosciences, Newcastle University, Newcastle upon Tyne NE4 5PL, U.K.

**Keywords:** autophagy, amino acids, lysosome, mTOR

## Abstract

Cells and organisms must coordinate their metabolic activity with changes in their environment to ensure their growth only when conditions are favourable. In order to maintain cellular homoeostasis, a tight regulation between the synthesis and degradation of cellular components is essential. At the epicentre of the cellular nutrient sensing is the mechanistic target of rapamycin complex 1 (mTORC1) which connects environmental cues, including nutrient and growth factor availability as well as stress, to metabolic processes in order to preserve cellular homoeostasis. Under nutrient-rich conditions mTORC1 promotes cell growth by stimulating biosynthetic pathways, including synthesis of proteins, lipids and nucleotides, and by inhibiting cellular catabolism through repression of the autophagic pathway. Its close signalling interplay with the energy sensor AMP-activated protein kinase (AMPK) dictates whether the cell actively favours anabolic or catabolic processes. Underlining the role of mTORC1 in the coordination of cellular metabolism, its deregulation is linked to numerous human diseases ranging from metabolic disorders to many cancers. Although mTORC1 can be modulated by a number of different inputs, amino acids represent primordial cues that cannot be compensated for by any other stimuli. The understanding of how amino acids signal to mTORC1 has increased considerably in the last years; however this area of research remains a hot topic in biomedical sciences. The current ideas and models proposed to explain the interrelationship between amino acid sensing, mTORC1 signalling and autophagy is the subject of the present review.

## Introduction

Sensing of the environment is essential for cells and organisms to coordinate their metabolic activity. Nutrients, growth factors and cellular energy trigger biosynthetic pathways and thereby stimulate growth. However, in response to nutrient limitation and other cellular stresses, cells induce a variety of responses that enable them to survive upon unfavourable conditions. Central amongst these is macroautophagy (hereinafter referred to as autophagy), a major intracellular degradation pathway that supports cell survival by eliminating damaged and potentially harmful cellular structures, and sustains energy homoeostasis during starvation by recycling of cytosolic components to compensate for nutrient deprivation [1].

**Figure 1:**
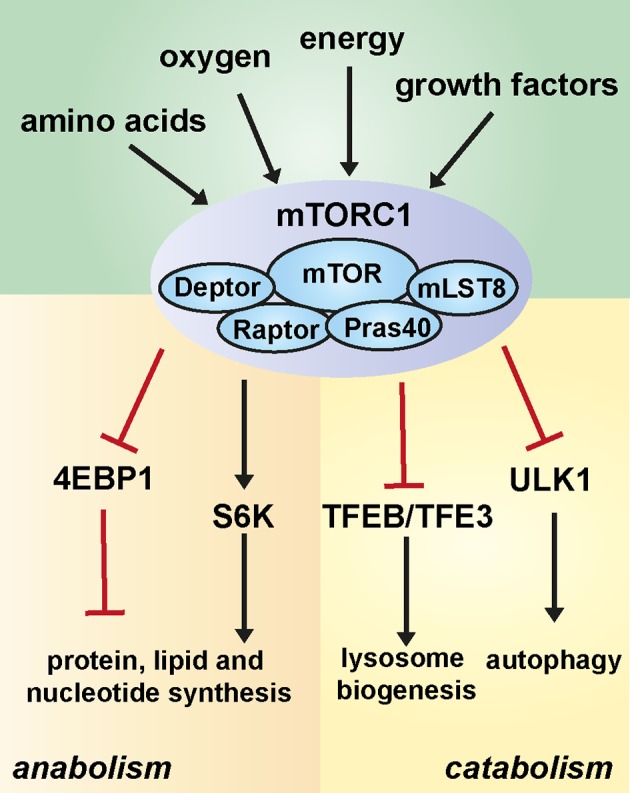
mTORC1 signalling links cellular growth with autophagy A range of physiological signals affects the activation status of mTORC1, including growth factor signalling, cellular energy levels via AMP-activated kinase (AMPK), oxygen levels and nutrients, particularly amino acids. The activation of mTORC1 regulates a number of cellular processes that affect the metabolic state of the cell. Through different mechanisms, mTORC1 signalling inhibits autophagy while promoting cell growth by stimulating biosynthetic pathways, including the synthesis of proteins, lipids and nucleotides.

The convergence point of anabolic and catabolic processes is the mechanistic target of rapamycin (mTOR), which senses fluctuations in extracellular and intracellular nutrients to modulate cellular growth, metabolism and survival. mTOR is an evolutionarily conserved serine/threonine protein kinase that belongs to the PI3K-related kinase (PI3KK) superfamily. This atypical kinase nucleates two structurally and functionally different complexes termed mTOR complex 1 (mTORC1) and mTOR complex 2 (mTORC2) [2]. In addition to the mTOR catalytic subunit, mTORC1 consists of regulatory-associated protein of mammalian target of rapamycin (Raptor) (a scaffold protein that is required for the correct subcellular localization of mTORC1) [3,4], mammalian lethal with Sec13 protein 8 (mLST8, also known as GßL) (which associates with the catalytic domain of mTOR and stabilizes the kinase activation loop) [3–6], and the two inhibitory subunits proline-rich Akt substrate of 40 kDa (PRAS40) [7–10] and DEP domain containing MTOR-interacting protein (DEPTOR) [11]. mTORC2 shares some components with mTORC1 including the core kinase protein mTOR as well as mLST8/GβL and DEPTOR. However, instead of Raptor, mTORC2 contains rapamycin-insensitive companion of mTOR (Rictor) and the regulatory subunits Sin1 [12–14] and Protor 1/2 [15,16]. mTORC1 and mTORC2 can be distinguished on the basis of their sensitivity to rapamycin which only inhibits mTORC1 [17]. The two complexes are responsive to different signals and produce different downstream outputs. While mTORC2 regulates cytoskeleton organization and cell survival [18,19], the major cellular role of mTORC1 is the control of cell growth. mTORC1 senses and responds to fluctuations in the levels of intra- and extracellular nutrients, primarily amino acids as well as growth factor signalling, cellular energy (via AMP-dependent kinase, AMPK) and oxygen levels. When active, mTORC1 promotes anabolic processes such as protein, lipid and nucleotide synthesis through phosphorylation of its downstream effectors ribosomal protein S6 kinase (S6K) and eukaryotic translation initiation factor 4E-binding protein (4E-BP) thus inducing cell growth and proliferation. At the same time it represses catabolic programmes via unc-51-like autophagy activating kinase 1 (ULK1), thus leading to the inhibition of autophagy. However, upon stress conditions (including amino acid deprivation) mTORC1 inactivation stimulates the formation of the ULK1-containing pro-autophagic complex, which ultimately entails the formation of autophagosomes [20–22] (Figure 1).

A master activator of mTORC1 kinase activity is the small GTPase Rheb (Ras-homologue enriched in brain) [23,24]. This GTPase has a C-terminal CaaX domain that is a subject to farnesylation which defines its subcellular localization in association with endomembranes, most importantly lysosomes [25,26]. The GTP-loaded Rheb interacts with the mTOR catalytic domain and activates mTORC1 [27]. To date, the regulators of GTP loading onto Rheb as well as the precise mechanism via which Rheb activates mTORC1 have yet to be discovered [28]. However, Rheb is negatively controlled by a heterotrimeric complex consisting of tuberous sclerosis complex 1 (TSC1), TSC2 and TRE2–BUB2–CDC16 domain family member 7 (TBC1D7) [29–31]. Specifically, the TSC2 subunit acts as a GTPase-activating protein (GAP) towards Rheb and promotes the hydrolysis of Rheb-bound GTP, thus converting Rheb to GDP-bound inactive form, which subsequently inhibits mTORC1 [23,24,29,32,33]. The TSC complex has emerged as a hub for numerous incoming cellular signals to regulate mTORC1 activity ranging from growth factor signalling, hypoxia, DNA damage or energy status [34,35]. Amongst them, PI3K/Akt (activated in the presence of growth factors), AMPK (which is activated in response to energy or glucose deprivation), the hypoxia-induced REDD1 (regulated in development and DNA damage responses 1), Erk (extracellular-signal-regulated kinase) [36] or GSK-3β (glycogen synthase-3β), all of which regulate TSC2 activity [34]. Despite the importance of the TSC complex in mTORC1 activity modulation, not all the inputs into mTORC1 signalling impinge directly on the control of the axis TSC2–Rheb. Indeed, amino acids, which are necessary and sufficient for the basal activation of mTORC1, can modulate mTORC1 activity via different mechanisms.

## Amino acids and mTORC1

Amino acids are fundamental nutrients that serve as building blocks for protein synthesis and cell growth. They are important metabolic intermediates that contribute to multiple cellular functions. Indeed, the availability of intracellular amino acids is essential to promote anabolic processes in the cell, including energy production, mRNA translation and, subsequently, cell growth and proliferation. When amino acids are scarce, protein synthesis is switched off and catabolic processes are triggered in order to generate amino acid sufficiency and restore cell function [37,38]. Thus, amino acids are both necessary and sufficient to have an impact on cellular metabolism with important implications for mTORC1 activity and autophagy regulation. Signalling via mTORC1 regulates expression of genes involved in glycolysis, pentose phosphate pathway, lipid biogenesis and pyrimidine biosynthesis [39,40]. Both influx of nutrients from the extracellular environment and recycling of intracellular resources regulate intracellular levels of amino acids to ensure the availability of free amino acids at an appropriate concentration for promoting anabolic processes in the cells. Cellular demands for amino acids are cell- and tissue-specific; while glutamine is an important energy source feeding the tricarboxylic acid (TCA) cycle particularly in glycolysis-dependent tumour cells [41], arginine influx and arginase levels are tightly regulated in liver for the urea cycle [42].

Leucine, glutamine and arginine have been widely shown as the main contributors to mTORC1 activation [43–47]. In particular, glutamine, in addition to serving as a key energy source, cooperates with leucine to activate mTORC1. Glutamine can be deaminated to produce α-ketoglutarate, which is both an intermediate in the TCA cycle and a regulator of mTORC1 activity and autophagy. However, this reaction requires the presence of leucine, which acts as a cofactor of the enzyme that catalyses the last step of glutaminolysis [48].

The mechanisms via which amino acids can influence mTORC1 are still only partially elucidated but the existing body of data suggests a highly complex control of amino acid sensing as described in the following sections.

## Lysosome as a hub of amino acid sensing

The first indication that amino acids play an essential role in the regulation of mTORC1 and that this signalling is connected to the regulation of autophagy was shown in a study of hepatocyte autophagy. Bloomaart and colleagues observed that, addition of amino acids inhibited autophagic proteolysis and this inhibition was correlated with the stimulation of S6 phosphorylation [49]. In addition, rapamycin treatment inhibited the effect of amino acids. This study was supported by the subsequent observation that treatment of the cells with protein translation inhibitors, which increase the pool of intracellular amino acids, activates mTORC1 and inhibits autophagy even in starvation conditions [50,51]. Nevertheless, the mechanism by which amino acids stimulate mTORC1 activity and inhibit autophagy and whether all amino acids or amino acids byproducts are sensed are still to some extent, an enigma. Leucine, glutamine and arginine have been well documented as the most important mediators in mTORC1 activation; however, no individual amino acid is sufficient for its activation in cells deprived of the remaining amino acids and therefore autophagy inhibition.

A key breakthrough in the understanding of the subcellular control of mTORC1 by amino acids was the finding that amino acids are able to regulate the intracellular localization of mTORC1 [26,52]. Specifically, upon amino acid withdrawal mTORC1 can be found diffuse throughout the cytoplasm, while replenishment of amino acids rapidly translocates mTORC1 to the surface of vesicles positive for Rab7 and LAMP2 (lysosome-associated membrane protein 2) [52], both markers for late endosomes and lysosomes. These observations suggested that amino acids may stimulate mTORC1 activity by translocating it to the lysosomal surface where it is presumed to interact with the small GTPase Rheb [26]. Subsequent studies have shown the scaffold protein Raptor as the mediator in the localization of the complex to the lysosome [52]. Tethering of mTORC1 to the lysosomal membrane is fundamental in the modulation of Rheb/TSC-mediated mTORC1 activation, thus providing a mechanism that explains why activation of mTORC1 by growth factors requires the presence of amino acids. These observations also identify the lysosome as a hub controlling mTORC1 activity. However, the fact that leucine can regulate mTORC1 activity independent of its effect on the lysosomal localization of mTOR illustrates an important distinction between the signals exerted by a single amino acid which strongly regulates mTORC1 and by a mixture of other amino acids [53]. These findings also establish that the sensing of leucine and the activation of mTORC1 have to be considered as complex processes which likely involve additional compartments besides the lysosome. Although Rheb is essential for amino acids to activate mTORC1, the primary amino acid sensing pathway appears to function in parallel to Rheb but involves a separate set of small GTPases, the Rag GTPases [26,54]. The Rag GTPases are members of the Ras superfamily of Rag GTPases and drive the recruitment and retention of mTORC1 to the lysosome upon amino acid availability [52]. The Rag GTPases are stably anchored to lysosomal membranes and act as docking sites for mTORC1 at this organelle by directly binding Raptor. Four members of this family have been described in mammals: RagA and RagB (RagA/B), which are highly homologous and functionally redundant, and RagC and RagD (RagC/D), which have also high sequence similarity and are functionally equivalent. RagA/B binds to RagC/D to form heterodimeric complexes and this dimerization is important for Rag protein stability but also essential for mTORC1 activation [26,54]. The association between mTORC1 and Rag GTPases is highly dependent on the guanine-nucleotide binding state of the heterodimer, with mTORC1 binding predominantly to heterodimers consisting of RagA/B·GTP and RagC/D·GDP. Amino acid sufficiency promotes the accumulation of RagA/B·GTP–RagC/D·GDP, allowing them to bind Raptor and recruit mTORC1 to the lysosomal surface, where it binds Rheb to form an active holoenzyme [52]. However, the absence of amino acids switches the Rag heterodimer into an inactive conformation containing GDP-bound RagA/B, thereby, releasing mTORC1 from the lysosomes [55,56]. This regulatory role of the Rags has been shown to be conserved in mice [57], flies [54] and yeasts [58]. Indeed, in both mammalian and *Drosophila* cells, overexpression of GTP-bound mutants of RagA or RagB renders mTORC1 constitutively active and insensitive to amino acid starvation [26,54]. As the amino acid-dependent mTORC1 signalling appears to be regulated by GTP/GDP charging of the Rag heterodimers, the study of the mechanisms that communicate amino acid availability to the Rag GTPases and the proteins involved in the modulation of their nucleotide-binding status has become of particular interest in the uncovering of amino acid sensing. Indeed, several guanine nucleotide exchange factors (GEFs) and GAPs that regulate the nucleotide status of the Rag GTPases have already been identified, with the most notable GEF being the Ragulator complex [52,59] and with GAPs including the GAP activity towards the Rags (GATOR) 1 complex (towards RagA/B) [55], the tumour suppressor folliculin (FLCN) in complex with FLCN-interacting protein 1/2 (towards RagC/D) [60] and leucyl tRNA synthetase (towards RagD) [44,61]. Furthermore, an arsenal of additional proteins has recently been identified as regulators of the Rag GTPases. Together, these proteins cooperate to ensure the right conformation of the Rag GTPases to promote mTORC1 localization and activity.

### Ragulator, a GEF for RagA/B

The multi-protein complex called Ragulator or late endosomal/lysosomal adaptor, MAPK and mTOR activator 1 (LAMTOR), has been shown responsible for both the regulation of the nucleotide-binding state of the Rag GTPases and their subcellular localization [52,59] since the Rag proteins do not contain lipid modifications to anchor them to the lysosomal membrane. The Ragulator complex comprises p18 [encoded by LAMTOR1 (late endosomal/lysosomal adaptor, MAPK and mTOR activator)], p14 (encoded by LAMTOR 2), MP1 (MEK-binding partner 1, encoded by LAMTOR 3), C7orf59 (encoded by LAMTOR 4) and HBXIP (hepatitis B virus X-interacting protein, encoded by LAMTOR 5). Amongst its components, LAMTOR 1 functions as a scaffold for the Rag GTPases and the two heterodimers LAMTOR2/LAMTOR3 and LAMTOR4/LAMTOR5 anchoring the complex to the lysosomal surface via dual N-terminal lipid modifications (myristoylation and palmitoylation) [62]. Although Ragulator orthologues have not been identified in yeast, a recent study unveils the ternary complex EGO 1–3 analogously localizing GTRs and TORC1 at the vacuolar surface [63]. Depletion of Ragulator components disrupts Rag GTPases attaching to the lysosome and prevents mTOR shuttling to its surface [52,59]. As discussed above, Ragulator is required for the localization of the Rag heterodimer to the lysosomal membranes and acts as a GEF towards RagA/B, promoting the exchange of GDP for GTP and consequent activation of the Rag complex and lysosomal recruitment of mTORC1 [59]. The GEF activity of the Ragulator complex appears to be restricted to RagA/B, not displaying any activity towards RagC/D or other GTPases [59] and all the components of the complex are essential for its GEF activity and thus, for amino acid sensing to mTORC1.

### GATOR complex, a GAP for RagA/B

GATOR is a multi-protein complex composed of two subcomplexes, GATOR1 and GATOR2, localized at the lysosomal membrane [55,56]. GATOR1 contains three proteins, DEP domain containing 5 (DEPDC5), nitrogen permease regulator 2-like protein (NPRL2) and NPRL3, whereas GATOR2 is composed of five components MIOS, WDR24, WDR59, SEH1L and SEC13. Of these two complexes, only GATOR1 interacts directly with RagA/B acting as a GAP thus leading to its deactivation and subsequently suppression of mTORC1 activity [55,56]. A recent study has characterized the multi-protein complex KICSTOR responsible for targeting GATOR1 to the lysosomal surface [64]. GATOR2 negatively regulates GATOR1 and relieves mTORC1 from its inhibition, thus rendering mTORC1 unresponsive to amino acids [55,56]. The inhibitory effect of GATOR2 on GATOR1 is mediated by Sestrin proteins (see below); however, how GATOR2 mediates its inhibiting effect and how amino acids regulate the complex is not fully settled.

### SH3BP4, a GAP for RagB

SH3 (Src-homology 3 domain)-binding protein 4 (SH3BP4) was found to reduce mTORC1 signalling by increasing both RagB GTP hydrolysis and preventing RagB GDP dissociation [65]. Its effect on mTORC1 signalling is more similar to that of a modulator.

### FLCN and LRS: GAPs for RagC/D

Folliculin (FLCN) is a GAP that activates mTORC1 by specifically regulating the nucleotide status of RagC/D. Its binding to the lysosome further requires association with the FLCN interacting proteins 1 and 2 (FNIP1 and 2) [60,66]. *In vitro* studies established that FLCN localizes to the lysosome upon amino acid starvation and only when RagA/B is GDP-loaded. GTP loading of RagA/B in the presence of amino acids stimulates the GAP activity of FLCN towards RagC/D and liberates FLCN from the lysosome [60].

The enzyme responsible for loading tRNA with leucine, leucyl tRNAsynthetase (LRS), has been reported as a positive regulator of mTORC1 activity by acting as a GAP for RagD [44,61]. LRS was found to translocate to the lysosome and bind Rag GTPases in a leucine-dependent manner where it acts as a GAP to promote the GTPase activity of RagD, required for mTORC1 activity [44]. However, in a subsequent study, the GAP activity of purified LRS towards RagD could not be reproduced [60] and the relevance of LRS to leucine sensing by mTORC1 remains to be validated.

### RNF152 and SKP2, E3 ubiquitin ligases for RagA

It has recently been reported that the ubiquitination of RagA by two independent E3 ubiquitin ligases, RNF152 [67] and SKP2 [68], negatively regulates mTORC1 activation. These studies revealed that targeting RagA for ubiquitination increases the interaction of RagA and GATOR1. Furthermore, overexpression of RagA ubiquitination-deficient mutant reduces RagA–GATOR1 interaction and thus increases mTORC1 activity. Deng et al. showed that amino acid withdrawal increases the RNF152–RagA interaction and, consequently, RagA ubiquitination [67]. However, Jin et al. proposed that amino acids promote SKP2-mediated RagA interaction with GATOR1, which is inconsistent with previous studies showing that amino acid starvation increases the interaction between RagA and GATOR1 [68]. To explain this controversial observation they propose a negative feedback mechanism whereby SKP2-mediated RagA ubiquitination recruits GATOR1 to restrict mTORC1 signalling upon sustained amino acid stimulation, which serves to maintain proper cellular functions.

## Mechanisms of amino acid sensing

**Figure 2:**
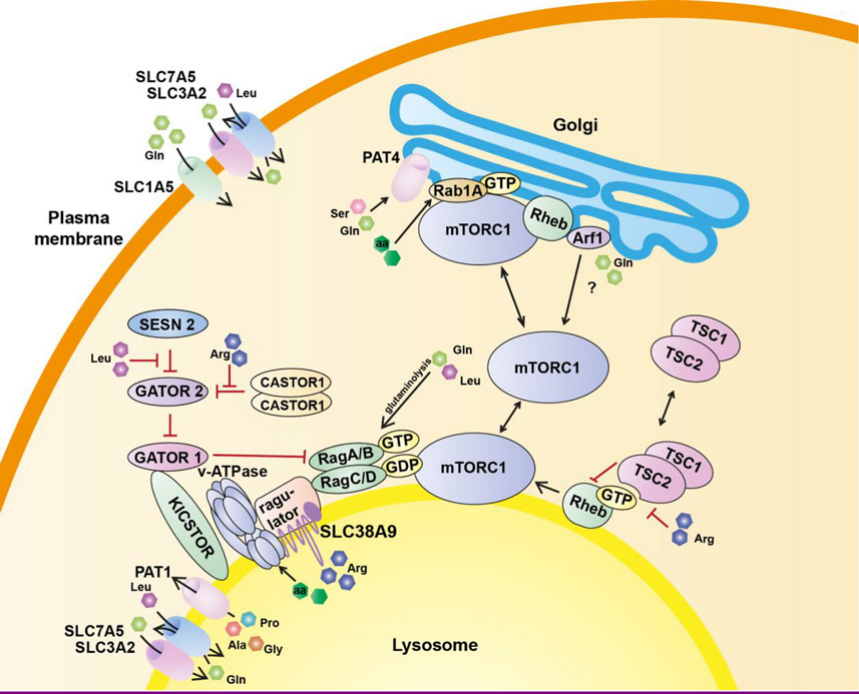
Models of amino acid-dependent mTORC1 regulation The presence of free amino acids is essential for the activation of mTORC1. Leucine, glutamine and arginine signal to mTORC1 by different mechanisms. These include cytoplasmic sensors, amino acid transporters and v-ATPase on the lysosomal membrane. The amino acid transporters sense specific amino acids in different subcellular compartments and act in cooperation with cytosolic amino acid sensors to control the activity of mTORC1. Other transporters act as conduits bringing amino acids inside the cell. All these mechanisms work to control the nucleotide-loading status of either Rag GTPases or Rheb, the most proximal regulators of mTORC1. mTORC1 hubs controlled by Arf1 and Rab1A/PAT4 appear to be Rag independent and are therefore not under the control of the cytosolic sensors. Arf1 is typically found on Golgi membranes but is reported to control a lysosomal sensing complex.

The findings described above identify the molecular platform necessary for mTORC1 activation at the lysosomal surface and have helped in the understanding of the amino acid-dependent Rag-mediated regulation. However, increasing evidence suggests other pathways to positively regulate mTORC1 in response to amino acids. Indeed, it is now clear that mTORC1 senses both, intralysosomal and cytosolic amino acids through distinct mechanisms (Figure 2). All these mechanisms cooperate to provide a sensory readout from more than one subcellular compartment with varying sensitivity to amino acids, thus leading to the tight control of the metabolic state.

### Lysosomal amino acid sensing

The identification of the lysosomal proton pump, the v-ATPase, as part of the lysosomal mTORC1 supercomplex, led to propose a mechanism of amino acid sensing where the v-ATPase acts as a sensor [69]. In addition to its role in proton pumping inside the lysosome, the v-ATPase responds to an increase in the intralysosomal, rather than cytosolic, amino acid concentration with a conformational change that weakens its binding with Ragulator [69]. These amino acid-sensitive interactions are required for proper nucleotide loading of the Rag GTPases, recruitment of mTORC1 to the lysosome and, thereby, activation of mTORC1 [69]. These observations led Sabatini and colleagues to propose the lysosome-centric “inside–out” model of amino acid sensing in which amino acids must accumulate in the lysosomal lumen to initiate signalling and the v-ATPase may act as a sensor [69].

However, the precise mechanism by which the v-ATPase signals amino acid sufficiency still remains to be determined; particularly the role of the ATPase activity for mTORC1 activation is not well understood. While chemical inhibition of ATP hydrolysis activity renders mTORC1 inhibition [69], other study has shown that the ATPase activity increases upon amino acid withdrawal [70]. Specifically, amino acid deprivation may drive conformational changes in the v-ATPase [69], potentially increasing its assembly [70] and consequently enhancing its interaction with the Rag GTPases–Ragulator complex. Moreover, others have demonstrated that intraluminal pH of the lysosome is important to support mTORC1 activity [71].

It also remains unclear whether amino acid sensing via the lysosomal lumen involves the direct trafficking of amino acids from the cytoplasm into the lysosome or whether the amino acids sensed here are generated as a consequence of the degradative process in the lysosome. Amino acid transport and accumulation within the lysosomal lumen do not appear to be driven by a proton gradient, as shown by freely diffusible alcohol ester derivatives of amino acids which enter the lysosome regardless of the v-ATPase activity [69,72]. Overexpression of the proton-assisted amino acid transporter (PAT) 1, which exports amino acids from the lysosome, inhibits mTORC1 activation [69], likely due to the leak of amino acids from the lysosome. However, these results require careful interpretation as overexpression of PAT1 can either promote growth and mTORC1 signalling *in vivo* and in cell culture [73,74] or inhibit these processes when expression levels are raised further, potentially via a dominant negative mechanism [69,73]. These observations indicate the importance of a careful balance in amino acid distribution or transport for proper mTORC1 activity.

Lysosomal localization of amino acid transporters led to a model in which amino acid sensing is carried out by these transporters based on their ability to bind amino acids from the lysosomal lumen and export them to the cytosol [75,76]. However, it is still unknown whether this system allows to sense specific, individual amino acids or overall concentration of amino acids. To date, the only transporter implicated in sensing of a specific amino acid from within the lysosome is the sodium-coupled amino acid transporter SLC38A9, which participates in sensing arginine following starvation and refeeding [77]. This observation is of particular interest since this transporter has higher affinity for other amino acids such as glutamine [78]. SLC38A9 has been identified as a physical and functional component of the Rag–Ragulator complex in lysosomes [77–79]. Its overexpression caused sustained mTORC1 activation even during amino acid starvation, while mTORC1 activation was abolished in SLC38A9 knockout cells [79]. Interestingly, knockdown of SLC38A9 prevents starvation-induced relocalization of mTOR to the cytoplasm [79]; however, the mechanism of mTOR retention is unknown.

PAT1, a member of the PAT or SLC36 family broadly expressed in humans, was shown to be required for amino acid-dependent mTORC1 activation and cell proliferation in embryonic kidney and breast cancer cells [74]. Interestingly, PAT1, as well as other members of this family, has been shown to transport alanine, glycine and proline [80,81], which are not potent mTORC1 activators, so it is not necessarily clear exactly how these transporters may influence mTORC1. PAT1 is concentrated at the surface of late endosomes and lysosomes in many cell types, wherein it forms a complex with Rag GTPases and has been suggested to mediate the regulation of amino acid-dependent mTORC1 localization [82]. Interestingly, this study highlights the cooperation of growth factors and amino acids in mTORC1 regulation, since PI3K/Akt/Rheb signalling increases lysosomal localization of PAT1 which would facilitate mTORC1 activation [82]. A recent work reports that accumulation of PAT1 in the lysosomal surface can be prevented by FLCN. Specifically, elevated FLCN levels lead to the decrease in PAT1 localization to the lysosome thus enabling cells to sequester amino acids such as leucine within the lysosome and activate mTORC1 even under amino acid starvation conditions [83]. This is an interesting concept, as FLCN is normally accumulated in the lysosome upon amino acid starvation. However, one explanation is that endogenous levels of FLCN are not sufficient to affect PAT1 and intracellular amino acid levels, and thus, mTORC1 is switched off [66].

The above-mentioned lysosomal amino acid transporters provide an arguably logical explanation that amino acids exported from the lysosome to the cytoplasm would be sensed by mTORC1, though the precise mechanism via which these transporters contribute to mTORC1 activation and autophagy suppression is still unclear. One interesting avenue is the concept of a dual-function amino acid transporter/receptor, which proposes that they can act as transceptors, functioning via sensing and signalling amino acid availability rather than their transport [73,84]. This may also help to explain the potent effect of PAT1 in cellular growth when it transports amino acids that are not potent mTORC1 activators [80,81] and why SLC38A9 senses arginine when it has higher affinity for glutamine [85].

Understanding how the transport of lysosomal amino acids is orchestrated is an area of significant interest for future research. Perhaps, the most straightforward explanation for the apparent complexity of the molecular machinery involved in this processes is that both lysosomal amino acid transporters and the v-ATPase cooperate to ensure tight control of the amino acid sensing at the lysosome. Supporting this idea, v-ATPase inhibition in HEK293T cells overexpressing SLC38A9 does not inhibit mTORC1 signalling upon amino acid deprivation, though inhibition of v-ATPase activity in cells not overexpressing SLC38A9 inhibits mTORC1 activation in response to amino acid stimulation [78]. Another recent study argues that the coupling of v-ATPase and PAT1 could explain why the reduction in the proton gradient across the lysosomal surface does not block amino acid-dependent mTORC1 relocalization [82]. The authors propose a model where PAT/Rag/Ragulator–v-ATPase complex is required to establish a microenvironment for cycling of protons and export of amino acids that is needed to drive amino acid sensing at the lysosome [82].

It is also of interest that the mechanisms that target amino acids to the lysosomes are largely unknown. To date, only one functional amino acid transporter, the heterodimeric transporter SLC7A5 (LAT1)/SLC3A2 that couples the import of leucine with the simultaneous efflux of L-glutamine and stimulates mTORC1 activation via v-ATPase, has been identified at the lysosome. SLC7A5/SLC3A2 is recruited to the lysosomal membrane via a protein called LAPTM4b [86].

The lysosomal lumen environment, therefore, provides a complex readout of amino acid levels for mTORC1 and uncovering further mechanisms that regulate their sensing will greatly enhance our understanding of this signal transduction pathway.

### Cytosolic amino acid sensing

Although it has been well documented that lysosomes are essential organelles for amino acid sensing, increasing evidence suggest that they can also signal to mTORC1 from the cytoplasm. Especially, some of the main contributors to mTORC1 activation, such as leucine, glutamine and arginine [43–45,87,88], are also sensed outside the lysosome. Cytoplasmic sensors of leucine and arginine have been recently identified. Sestrin (SESN) 2, which has long been known to influence cellular growth and metabolism, acts as a direct leucine sensor. Structural and biochemical analyses established that SESN2 can bind and inhibit GATOR2 function under leucine deprivation conditions, and dissociates from it upon leucine binding [89,90]. Moreover, a recent study revealed that SESN2 expression is induced upon long-term starvation via the stress-responsive transcription factor ATF4 [91], suggesting a novel role for SESN2 as an indirect mediator of prolonged amino acid starvation.

Using a similar mechanism, cytosolic arginine activates mTORC1 by directly binding the recently identified arginine sensor CASTOR1 (cellular arginine sensor for mTORC1). This interaction disrupts the CASTOR1–GATOR2 complex, which enables GATOR2 to inactivate GATOR1, thus leading to increased Rag-dependent mTORC1 signalling [89,92]. These findings establish GATOR2 as a central node in the signalling of both leucine and arginine to mTORC1, though the molecular function of GATOR2 and the mechanisms through which SESN2 and CASTOR1 regulate it still remain elusive.

Interestingly, although the TSC2/Rheb signalling input has classically been considered to be insensitive to amino acids [27,93,94], recent studies propose the ability of amino acids to regulate the localization of the TSC complex and consequently, mTORC1 activity. These findings imply a mechanism via which amino acids can control the activation of mTORC1 by growth factors [85]. Particularly, arginine suppresses lysosomal localization of the TSC complex and its interaction with Rheb. By interfering with TSC–Rheb complex, arginine relieves allosteric inhibition of Rheb by TSC [43]. These findings highlight the cooperation of amino acids with other nutrient inputs (i.e. growth factors) in the activation of mTORC1.

The metabolism of glutamine can also activate mTORC1. Specifically, the production of α-ketoglutarate (α-KG) via glutaminolysis stimulates GTP loading of RagB [87]. This process, which takes place in mitochondria, is supported by leucine availability which acts as a cofactor for the enzyme glutamate dehydrogenase that participates in deamination of glutamine [87]. The glutaminolysis model provides a sensing mechanism for the activation of mTORC1 by leucine and glutamine availability.

### Amino acid sensing from the plasma membrane

In parallel with the identification of intracellular mediators of amino acid sensing discussed above, other interesting avenue for this research is focused on finding cell surface molecules involved in the uptake of extracellular amino acids. Very recently it has been reported that the G-protein coupled taste sensing receptor TIR1/TIR3 is an early sensor of extracellular amino acid availability [95]. Reduced expression levels of TIR1/TIR3 impaired the activation of mTORC1 by amino acids, caused mislocalization of mTORC1 and accelerated autophagy even under nutrient-rich conditions [95]. It has been proposed that TIR1/TIR3 activation increases Ca^2+^ influx, thus activating Erk1/2 [95] which ultimately activates mTORC1 by phosphorylating Raptor and TSC2. Although the contribution of Erk1/2 phosphorylation to mTORC1 activity via a direct mechanism is still debatable [96], a role for intracellular Ca^2+^ in mTORC1 activation has been demonstrated [95,96].

In addition to TIR1/TIR3, a number of solute carriers have been identified as mediators in modulating mTORC1 activity and autophagy, some of them belonging to families capable of transporting amino acids. These amino acid transporters were initially implicated in mTORC1 regulation as passageways through the plasma membrane, enabling amino acids to enter cells and activate cytoplasmic amino acid sensors [50]. Amongst them, the amino acid transporter, solute carrier family 1, member 5 (SLC1A5) regulates glutamine uptake and loss of its function inhibits cell growth and activates autophagy [97]. Another example is the above-mentioned heterodimer SLC7A5 (LAT1)/SLC3A2 which couples import of leucine with export of glutamine [97]. In fact, the glutamine transported into the cell by SLC1A5 can be used by SLC7A5/SLC3A2 to exchange with leucine, thus increasing intracellular leucine levels [97]. This could indicate the different impact of individual amino acids on mTORC1 – whilst leucine directly acts on the mTORC1 pathway glutamine appears to be a facilitator to maximally activate mTORC1. Likewise, SLC43A1 (LAT3) [98] and SLC38A2 (SNAT2) [99], both transporters of neutral amino acids, have also been linked to mTORC1 signalling. It is likely that, in addition to specific amino acids, a basal concentration of other amino acids is also required to activate mTORC1 [43]. Further work is likely to identify the mechanisms that regulate these amino acid transporters and therefore, contribute to controlling intracellular concentrations of amino acids and subsequently mTORC1. Indeed, the turnover of PAT4, which is regulated by the small GTPase Rab12, can impact on modulation of mTORC1 activity and autophagy possibly because its internalization in recycling endosomes (wherein PAT4 is not functional) consequently reduces the influx and therefore the intracellular levels of amino acids [100]. This observation highlights the trafficking and turnover of amino acid transporters as a mechanism of mTORC1 regulation. It is also possible that a dynamic relationship exists between plasma membrane and intracellular pools of amino acid transporters, and deciphering the mechanisms that regulate this cross-talk will further our understanding of amino acid sensing by mTORC1.

### Amino acid sensing from the Golgi

Two molecules involved in membrane trafficking have been identified as potent regulators of mTORC1. Thus, Arf1, classically implicated in Golgi transport, has been shown to participate in glutamine-dependent mTORC1 activation via a Rag-independent lysosomal mechanism in mammalian cells [45]. Additionally, Rab1A, previously known for its role in vesicle transport between ER and Golgi, is involved in amino acid-dependent mTORC1 activation on the Golgi, also in a Rag-independent fashion [101]. Specifically, Rab1A interacts with mTORC1 in response to amino acid sufficiency to promote the colocalization of mTORC1 with Rheb in Golgi [101]. The authors report the preferential binding of mTORC1 to GTP-bound Rab1A through Raptor, which is increased in the presence of amino acids, and they also show that the Rab1A–mTORC1 interaction requires the prenylation modification that tethers Rab1A to the Golgi. These findings place Rab1A, mTOR, Raptor and Rheb at the Golgi following amino acid stimulation. Knockdown of Rab1A disrupts mTORC1 localization to the Golgi, but not to lysosomes, supporting a model in which an mTORC1 hub is assembled on Golgi membranes. Based on these findings and the reported localization of TSC to the Golgi in addition to lysosomes [102], the authors contend that Rab1A might function in an amino acid sensing pathway distinct from the classical Rag pathway that either is redundant or senses a different pool of amino acids or subcellular cues. While lysosomal mTORC1 could detect extracellular or autophagy-derived amino acids in the lysosomal lumen, Golgi-localized mTORC1 could be regulated by amino acids trafficked back in a retrograde manner from the endosomal system or brought into the Golgi lumen by Golgi-localized amino acid transporters. As a matter of fact, PAT4 has been shown to be predominantly localized on the *trans*-Golgi network in several cell types where it interacts with mTORC1 and Rab1A, to control serine- and glutamine-dependent activation of mTORC1 [101,103].

Clearly more studies are required to clarify how different mechanisms of amino acid sensing at multiple intracellular compartments are integrated. One exciting possibility is that the intricate network of trafficking pathways between the Golgi, late endosomes/lysosomes and the plasma membrane allows activated mTORC1 to be transported within the endomembrane system and to find its substrates [104]. This possibility could explain the mTORC1 sensitivity to the activity of the regulators of vesicle trafficking such as Rab and Arf GTPases.

### Alternative amino acid sensing mechanisms

A number of other pathways have been shown to positively regulate mTORC1 in response to amino acids. Amongst them, inositol polyphosphate multikinase (IPMK) appears to be a cofactor involved in maintaining the binding of mTOR with raptor to supress mTORC1 activity upon amino acid deprivation, whereas the presence of amino acids converts this complex into a low-affinity state thereby allowing lysosomal relocation of mTORC1 for activation [105]. The action of IPMK on amino acid signalling to mTORC1 is independent of its catalytic activity. In addition, the mitogen-activated protein kinase 3 (MAP4K3) was shown to be required for amino acid-dependent mTORC1 signalling in both mammalian cells and flies [106,107], though its role in amino acid sensing is not entirely clear.

Another protein recently implicated in the activation of mTORC1 in response to amino acids is the human class III PI3K (phosphoinositide 3-kinase) hVps34, which catalyses the synthesis of the lipid, phosphatidylinositol (3)-phosphate (PI(3)P), an important regulator of endocytosis and autophagy. hVps34 is activated by amino acids via a calcium-dependent mechanism [96] and this leads to an accumulation of PI(3)P in cells, which is believed to cause the recruitment of proteins to early endosomes, forming an intracellular signalling platform that promotes mTORC1 activation [108,109]. This pathway appears to be vertebrate-specific as studies in flies have reported no observable effect of this protein on mTORC1 signalling [110].

Additionally, it has been demonstrated that the phosphatidic acid producing enzyme phospholipase D (PLD) is indispensable for lysosomal translocation and activation of mTORC1 in response of amino acids [111,112]. Specifically, PLD1 has been proposed to be part of the protein complex anchoring mTORC1 to the lysosomal membrane [112,113].

Finally, in addition to its role on autophagy, the autophagy receptor protein p62 (also known as sequestosome 1, SQSTM1) has recently been identified as an integral part of the mTORC1 complex. p62 interacts in an amino acid-dependent manner with TNF receptor associated factor 6 (TRAF6), mTOR and raptor and Rag GTPases to promote mTORC1 recruitment to lysosomes where it is activated. Specifically, p62 favours the formation of the active Rag heterodimer and, together with TRAF6 promotes mTORC1 translocation to the lysosome. Via an incompletely understood mechanism, TRAF6-dependent ubiquitination of mTOR is then required for amino acid-dependent activation of mTORC1 [114].

## Amino acid/energy sensing and autophagy–lysosome pathway

Autophagy is a multi-step process in which autophagosomes engulf cytosolic organelles and macromolecules and, through fusion with lysosomes, target their constituents for degradation [115]. The pathways and molecular mechanisms controlling autophagy are well studied. The autophagic machinery is evolutionarily conserved from yeast to mammals where a set of autophagy-related (Atg) proteins orchestrates distinct stages of the pathway, such as initiation, elongation, maturation and fusion. Autophagy occurs in the cell at a basal level in order to maintain energy homoeostasis and cellular functions. However, it can be further activated above basal levels in response to many cellular stressors such as oxygen, energy and nutrient (e.g. amino acid) deprivation [22].

It is now clear that cellular homoeostasis of metabolism and growth is exquisitely controlled by the coordination of AMPK and mTORC1. AMPK is crucial to the cellular response to low energy levels and, once active, drives processes which will replenish cellular energy stores, such as autophagy while inhibiting biogenic synthesis [116]. Opposite to AMPK, activated mTORC1 shifts the metabolic programme of the cell from catabolic to anabolic metabolism by up-regulating the synthesis of building blocks for cell proliferation, including proteins, lipids and nucleotides [117]. As both mTORC1 and AMPK are essential for the cell to maintain energy and nutrient homoeostasis, it has been appreciated that their signalling pathways must be coordinated. Several mechanisms of cross-talk have been identified. Thus, it was shown that AMPK inhibits mTORC1 by activating TSC2 [118–120] or by directly phosphorylating the mTORC1 component, Raptor on Ser722 and Ser792 [121]. Recent research has also revealed that the lysosome may function as a crucial hub for integration of signalling in response to both nutrients and energy by reporting the lysosomal v-ATPase–Ragulator complex as a common activator for AMPK and mTORC1 [122]. The first indication that AMPK might have a function on lysosomes was the identification of the interaction of its binding partner, AXIN, with one of the components of the Ragulator complex, LAMTOR1 [123]. Later studies revealed that AXIN functions as a scaffold protein for AMPK and its upstream regulator, the liver kinase B1 (LKB1), displaying a weak constitutive interaction with both AMPK and LKB1 which was found to be enhanced in the presence of AMP or upon glucose starvation [122]. Specifically, under energy stress, LKB1-bound AXIN translocates to the lysosomal surface wherein it interacts with the v-ATPase complex and the AMP-bound AMPK. It was also shown that the v-ATPase complex was required for the enhanced AXIN/LKB1–LAMTOR1 interaction under starvation [122]. The translocation of the complex LKB1–AXIN not only activates AMPK and turns on catabolic processes, but also turns off anabolic processes through facilitating starvation-induced lysosomal dissociation and inhibition of mTORC1. These observations support the role of the v-ATPase–Ragulator complex as a dual sensor for energy/nutrients and posit the lysosome as a hub coordinating AMPK and mTORC1 signalling [122].

Whereas the role of amino acids as key signal factors in the activation of mTORC1 has been accepted, to date, the inhibitory effect of amino acids on AMPK has been a debatable issue. Some studies have observed the inhibition of AMPK by amino acids [124–126], though this has not always been the case [127,128]. A recent work suggests amino acids as novel metabolic activators of AMPK. In this study, AMPK responded acutely and within 1 min to amino acid and its activation was independent of mTORC1 [129]. It should be noted that all previous studies observed AMPK phosphorylation at the earliest at 15 min post amino acid addition. This study explains that, at this time point, AMPK is repressed and postulates this as the reason why earlier studies may have missed the early AMPK induction by amino acids. Furthermore, they demonstrate that AMPK activation occurs via the Ca^2+^/calmodulin-dependent kinase kinase-β (CaMKKβ) and that activation of the CaMKKβ–AMPK axis by amino acids does not inhibit mTORC1 but sustains autophagy. They propose that this sustained autophagy under amino acid sufficiency might be required to maintain protein homoeostasis and deliver metabolite intermediates for biosynthetic processes [129].

Regulation of autophagy by TORC1 and AMPK signalling is best understood at the stage of autophagosome biogenesis. Autophagy pathway is mostly orchestrated by Atg proteins that nucleate distinct complexes, amongst which ULK1 and Vps34 complexes act as important gatekeepers for autophagy induction [130–134]. The ULK1 complex consists of three regulatory subunits Atg13, the focal adhesion kinase family interacting protein of 200KDa (FIP200), Atg101 and the core Ser/Thr kinase ULK1. ULK1 is essential for autophagy induced by amino acid starvation and is directly regulated by mTORC1 through phosphorylation at Ser757 [135,136]. The reversible phosphorylation of ULK1 is a central mechanism by which starvation-induced autophagy is regulated. In amino acid-rich conditions, mTORC1 binds to, phosphorylates and thereby inactivates ULK1 and Atg13, whereas on sensing a decrease in amino acid levels, mTORC1 dissociates from the ULK1 complex, thus relieving the inhibition of ULK1. Additionally, AMPK coordinates with ULK1 to regulate autophagy. AMPK promotes autophagy by directly phosphorylating several residues in ULK1 (including Ser555, Ser317 and Ser777, mouse ULK1 numbering) which leads to its activation [137–140]. Under nutrient sufficiency, high mTORC1 activity prevents ULK1 activation by phosphorylating ULK1 Ser757 and disrupting the interaction between ULK1 and AMPK. Interestingly, under starvation, ULK1 can phosphorylate all three AMPK subunits to down-regulate AMPK activity [141]. This coordinated phosphorylation shows a finely balanced regulatory signalling network [142].

It has been proposed that amino acid deprivation triggers more rapid autophagy than chemical inhibition of mTORC1. Indeed, in addition to the inhibition of mTORC1, amino acid starvation causes an increase in the phosphatase activity of PP2A towards ULK1 which triggers the rapid dephosphorylation of ULK1 and consequently, accelerates the autophagic response [143]. Specifically, amino acid withdrawal stimulates PP2A activity by inducing its dissociation from the inhibitory protein α4, which is believed to keep PP2A in an inactive state [143].

Another autophagic complex required for autophagy induction, the Vps34 complex which includes Atg14, Vps15 and Beclin-1, can be regulated by amino acid sufficiency. Upon amino acid/nutrient abundance, the mTORC1-dependent phosphorylation of Atg14-containing Vps34 complex inhibits the autophagosome biogenesis and suppresses autophagy. Conversely, depletion of amino acids causes mTORC1 inhibition, thereby relieving this pro-autophagy complex to stimulate autophagy [144]. Vps34 contributes to a variety of cellular functions and, as discussed above, this lipid kinase also participates in amino acid-dependent activation of mTORC1 [108,109]. Vps34 may exist (as in yeast) in two different populations, with and without Beclin-1 association [145].

**Figure 3:**
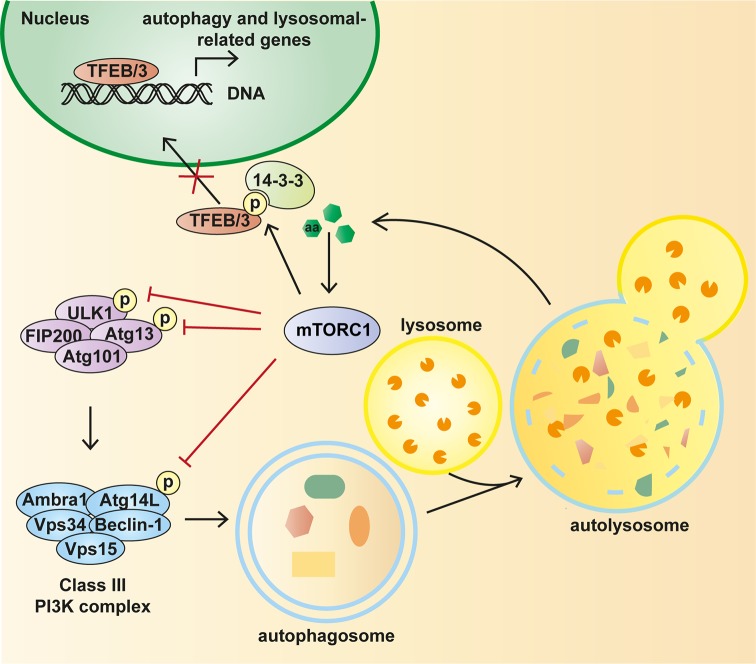
Amino acid-dependent regulation of autophagy by mTORC1 Schematic representation of the cellular signalling pathways governed by amino acids in the regulation of autophagy via mTORC1. Amino acid sufficiency activates the mTORC1 pathway which inhibits autophagy at multiple steps. Although the best-characterized mechanism via which mTORC1 inhibits autophagy involves the direct control of ULK1, mTORC1 also regulates Atg14-containing Vps34 complex and TFEB. In amino acid-rich conditions, mTORC1 binds to, phosphorylates and thereby inactivates the autophagy initiators ULK1 and Atg13, which are present in a complex with FIP200 and Atg101. Likewise, mTORC1 phosphorylates TFEB and TFE3 and this event facilitates the interaction between TFEB and TFE3 and the cytosolic chaperone 14-3-3 which retains them in the cytoplasm. In the absence of activating stimuli, autophagy is induced through the dissociation of mTORC1 from the ULK1 complex, thus relieving the inhibition of ULK1 which is then responsible of its own phosphorylation as well as phosphorylation of Atg13, FIP200 and Raptor. ULK1 is then able to activate this PI3K complex and promote autophagosome synthesis. In addition, mTORC1 inactivation leads to relocalization of TFEB and TFE3 to the nucleus where they cause the expression of multiple lysosomal and autophagy-related genes. This allows the cell to maintain a critical level of energy and metabolites for surviving the starvation condition.

In addition to autophagic complexes, amino acids also control autophagy through the family of transcriptional factors regulating autophagic and lysosomal gene expression (Figure 3). The prototypic member of this family is the transcription factor EB (TFEB) [146,147]. TFEB controls lysosomal biogenesis and autophagy by positively regulating genes belonging to the CLEAR (coordinated lysosomal expression and regulation) network [148–150]. Thus, TFEB activation leads to an increased number of autophagosomes and autophagic flux, biogenesis of new lysosomes, and clearance of storage material [146,149,151,152]. TFEB activity and its nuclear translocation correlate with its phosphorylation status [146,153]. Recent studies have reported that Rag GTPases and lysosomal mTORC1 regulate TFEB localization by phosphorylating the transcription factor on several serine and threonine residues, including Ser211, under nutrient-rich conditions [147,151]. TFEB phosphorylation on Ser211 facilitates its interaction with the cytosolic chaperone protein 14-3-3 which retains (and therefore inactivates) TFEB in the cytoplasm [154]. Under amino acid deprivation, mTORC1 inactivation leads to dephosphorylation and relocalization of TFEB to the nucleus where it promotes the expression of lysosomal and autophagy-related genes [146]. This cellular response allows the adaptation to diminished amino acid levels by increasing lysosomal and autophagic compartments in order to maintain the levels of energy and metabolites required to survive starvation condition.

Similarly, the transcription factor TFE3, which belongs to the same family that TFEB, is recruited to lysosomes through direct interaction with active Rags and this recruitment is critical to inactivate TFE3 under nutrient-rich conditions. Specifically, mTORC1 phosphorylates TFE3 at several residues but Ser321 is particularly relevant as this phosphorylation creates a binding site for the cytosolic chaperone 14-3-3 which sequestrates this transcription factor in the cytosol [147,154–157]. The inactivation of Rags and mTORC1 following starvation allows the transport of TFE3 to the nucleus and thus promotes cellular clearance [155]. TFEB and TFE3 are partially redundant in their ability to induce lysosomal biogenesis in response to nutrient starvation, but both must be present for a maximal response [155].

Besides the control that mTORC1 exerts on TFEB and TFE3 recruitment, a recent work has reported a feedback circuit wherein these transcription factors can control lysosomal recruitment of mTORC1 by regulating the expression of RagD [158]. The fine modulation of this circuit is essential for metabolic adaptation to nutrient availability**.** At the same time, the nuclear localization of the transcription factor zinc finger with KRAB and SCAN domains 3 (ZKSCAN3), which acts as a master repressor of lysosomal biogenesis and autophagy in fed cells, is also regulated by mTORC1 [159]. Overall, the emerging picture reveals the close collaboration between mTORC1 and several other master regulators of autophagy and lysosomal biogenesis in order to facilitate an efficient response to the varying nutrient demands of the cell [156]. As has been described in this section, amino acid starvation up-regulates autophagic and lysosomal activity through suppression of mTORC1-mediated phosphorylation of ULK1, Atg13, Atg4–Vps34, TFEB and TFE3 [135,136,139,147,155,156] (Figure 3). However, it has been recently shown that amino acid withdrawal, besides increased turnover of autophagic cargo, causes selective remodelling of the plasma membrane in yeast [160,161]. In this model system, endocytic trafficking of membrane proteins to the vacuole (the yeast equivalent of the lysosome) allows their hydrolysis into amino acids which provide material to fuel biosynthesis of more lysosomal proteins. These newly synthesized lysosomal proteins enhance hydrolysis of a later wave of autophagic cargo [161]. This starvation-induced protein turnover has been proposed to be regulated by TORC1. On one hand, TORC1 signalling promotes the endocytosis of certain plasma membrane proteins [162]. On the other hand, TORC1 inactivation triggers endocytosis of other plasma membrane proteins that are degraded in an endosomal sorting complex required for transport (ESCRT)-dependent manner via the multivesicular body (MVB) pathway [160,162–165]. Although it has been proposed that TORC1 signalling controls the major ubiquitin ligase required for endocytosis in yeast, Rsp5, how TORC1 signalling controls Rsp5-dependent cargo specificity and the selective ubiquitination of many different cargoes at the same time is currently not clear [161]. Furthermore, how the subsequent ubiquitin-dependent degradation of membrane proteins via the MVB pathway helps to meet the specific metabolic and energetic demands of the cell during nutrient limitation is not fully understood. The result of starvation-induced up-regulation of endocytosis and vacuolar degradation is a pool of amino acids that is essential for the adaptation to the lack of nutrients. Thus, the MVB pathway helps to maintain translation during acute starvation until autophagy, the long-term starvation–response pathway, is fully active. However, is not clear how selective (MVB) and non-selective (autophagy) lysosomal proteolysis pathways cooperate to mediate cell survival during nutrient limitation.

Autophagy is an important avenue for nutrient supply, particularly during amino acid starvation, which contributes amino acids to restore mTORC1 activity [166,167]. In turn, autophagy-mediated restoration of mTORC1 induces autophagy termination and reformation to lysosomes which complete the feedback loop [166]. Although it would be tempting to envisage that autophagy restores the amino acid pool to reactivate mTORC1, some studies have detected only a partial restoration of amino acid abundance by autophagy [166,168], which suggests the existence of a more precise mechanism. Specifically, an increase in the synthesis and release of glutamine during starvation has been reported. A recent work shows that glutamine is required for autophagy-induced mTORC1 reactivation during amino acid starvation and suggests that extracellular glutamine may function as specific starvation-induced nutrient signal to regulate mTORC1 [169]. In addition, this study demonstrates that the restorative effect of glutamine on mTORC1 signalling requires the conversion of glutamine to glutamate, which in turn sustains the non-essential amino acid pool via transamination [169].

## Challenges and future perspectives

Since amino acids are essential building blocks in the cell, the ability to regulate their levels is fundamental for cell survival, growth and development. The control of intake, utilization and recycling of amino acids is subjected to a fine regulation which involves a number of mechanisms that ensure amino acid availability in order to support cellular function. All these mechanisms integrate amino acid sufficiency with anabolic processes and autophagy. Given the massive impact of amino acids on autophagy and the importance of autophagy for cell function, the research in the field of amino acid sensing has expanded exponentially over the last years [170–172]. At the epicentre of amino acid sensing is the kinase mTORC1 that governs a rapid cellular response to fluctuations in amino acid levels. mTORC1 activity is inhibited upon amino acid withdrawal, which increases flux through autophagy pathway leading to recycling of amino acids and, thereby, restoration of mTORC1 activity together with the inhibition of autophagy. Thus, the study of the mechanisms via which mTORC1 senses amino acid levels has become fundamental to the understanding of amino acid signalling.

The discovery of the lysosomal Rag GTPases as important regulators of amino acid-dependent mTORC1 activation has focused most of the studies on the identification of molecules that could be involved in amino acid sensing by modulating the nucleotide state of the Rag GTPases. To date, one of the most relevant has been the characterization of the v-ATPase, which has led to the prediction that amino acids are sensed within the lysosome [69]. Nonetheless, besides the well-known role of lysosomes in amino acid sensing, it is now accepted that mTORC1 senses both intralysosomal and cytosolic amino acids through different mechanisms. From the plasma membrane to the cytosol, novel molecules directly involved in the sensing of amino acids have recently been revealed. Some examples are IPMK, MAPK43 or Vps34, which have been proposed as important intracellular proteins in amino acid signalling, although the mechanisms via which these molecules sense amino acids and activate mTORC1 still remain elusive. An exciting advance in the field represents the identification of specific cytoplasmic amino acid sensors for some of the most potent activators of mTORC1, leucine and arginine [90,92,173]. These findings leave open the question of the existence of additional amino acid sensors in the cytosol with specificity for other amino acids. Furthermore, intracellular amino acids exist as free forms or bound to proteins, lipids or other molecules. Future work will be needed to clarify whether the equilibrium between these different pools determine which amino acids are available to the sensors [172]. One particular aspect that will be of interest is the relative contribution of these different pools of amino acids in the activation of mTORC1.

An interesting avenue that is expanding in this field is the importance of extracellular amino acids in determining intracellular amino acid concentrations. This has led to the study of cell surface amino acid transporters since they are in an ideal location to relay nutritional information, as well as amino acids themselves, to the cell interior. Although these transporters were initially classified by their ability to translocate specific amino acids across the lipid bilayer, they have lately emerged as important amino acid sensors controlling mTOR recruitment and activation [73,171]. Indeed, increasing evidence exists suggesting that they can activate amino acid-dependent signalling either in the presence or absence of transport, by acting as transceptors [171]. This concept of dual-function suggests that part of the sophisticated control governing the trafficking of amino acids has to do with their signalling function rather than with the control of transport. Thus, for those amino acid transporters that act as sensors for mTORC1 future work is likely to unveil whether they directly interact with the amino acids they are believed to sense [171].

As shown by recent studies, amino acid transporters do not function exclusively at the plasma membrane; they can also be found intracellularly where they may increase local concentrations of amino acids. These findings, in addition to reinforce the role of lysosome as amino acid storage sites with high concentrations of amino acids at their surface, open an exciting field of research that predicts that amino acids could be sensed in other subcellular compartments. Thus, the concentration of amino acids may exist in gradients across the intracellular space, specifically within various organelles, and could differ significantly compared with those found in the cytosol since they contain a distinct set of amino acid transporters [172]. In this multi-hub sensing model, many signalling cascades controlled at a subcellular level could provide distinct readouts of the cell’s metabolic state and, thereby, the flexibility for a more refined response within a single cell.

Despite the explosion of knowledge in amino acid sensing in the last years, one major unsolved question in this field revolves around how these novel mechanisms fit with the model of Rag GTPases-dependent amino acid regulation. Furthermore, how the signals from intracellular nutrients and exogenous growth factors are integrated and what is the relative role of Rheb and Rag GTPases in this process remain the subject of further investigation [174,175]. Future work is likely to provide more information on the exquisite complexity of the signalling network of amino acids upstream of mTORC1 to define the exact roles and cooperation of these mechanisms in the tight control of cellular nutrient response pathways such as autophagy.

Finally, nutrient sensing pathways are of particular socio-economic significance as their perturbation is associated with the process of ageing and contributes to a number of age-related conditions including cancer, neurodegeneration and cardiovascular disease as highlighted in several reviews in this issue [182,183,184]. Thus, deregulation of mTORC1 has been described in cellular and organismal senescence where it can remain active even during nutrient deprivation [176–178]. This could drive uncontrolled growth, hypertrophy and chronic suppression of autophagy, all potential contributing factors in age-related pathology [179–181]. Further understanding of the molecular mechanisms of mTOR and autophagy and their deregulation in human pathology will facilitate identification of interventions aiming at the extension of healthy lifespan.

## Summary

- Amino acids have been elucidated as important regulators of the cell metabolism. As they are essential building blocks in the cells, organisms have developed a number of mechanisms to regulate their intracellular levels.
- mTORC1 governs the cellular response to fluctuations in amino acid levels, increasing flux through autophagy pathway when amino acids are scarce in order to generate amino acid sufficiency, in turn restoring mTORC1 activity and inhibiting autophagy.
- The mechanisms via which amino acids can influence mTORC1 are still partially elucidated demonstrating the complex control of amino acid sensing. The best-described amino acid sensing pathways involve the Rag GTPases, although a number of Rag-independent amino acid sensing mechanisms have recently been identified.
- Increasing evidence suggests that amino acids can signal from both the lysosomal lumen and the cytoplasm. Although the identification of the complex v-ATPase–Ragulator–Rag GTPase established the first evidence that amino acid sensing may propagate from within the lysosome (inside–out model), the recent discovery of cytoplasmic amino acid sensors suggests that some amino acids can also signal from the cytoplasm.
- Lysosomes, by providing a platform for Rag and Rheb GTPases, create a signalling hub that finely regulates mTORC1 and AMPK activities and downstream anabolic and catabolic processes including autophagy.
- Further identification of new molecular players in mTORC1 signalling will undoubtedly expand our understanding of the exquisite complexity of amino acid signalling upstream of autophagy and their perturbation in human diseases.

## Abbreviations

4E-BP: eukaryotic translation initiation factor 4E-binding protein
α-KG: α-ketoglutarate
AMPK: AMP-activated kinase
Atg: autophagy-related protein
CaMKKβ: Ca^2+^/calmodulin-dependent kinase kinase β
CLEAR: coordinated lysosomal expression and regulation
DEPDC5: DEP domain containing 5
DEPTOR: DEP domain containing MTOR-interacting protein
Erk: extracellular-signal-regulated kinase
ESCRT: endosomal sorting complex required for transport
FIP200: family interacting protein of 200 KDa
FLCN: folliculin
FNIP1/2: FLCN-interacting protein 1/2
GAP: GTPase-activating protein
GATOR1: GAP activity towards the Rags 1
GATOR2: GAP activity towards the Rags 2
GEF: guanine nucleotide exchange factor
GSK-3β: glycogen synthase kinase 3β
HBXIP: hepatitis B virus X-interacting protein
IPMK: inositol polyphosphate multikinase
LAMP2: lysosome-associated membrane protein 2
LAMTOR: late endosomal/lysosomal adaptor, MAPK and mTOR activator 1
LKB1: liver kinase B1
LRS: leucyl tRNAsynthetase
MAP4K3: mitogen-activated protein kinase kinase kinase kinase 3
mLST8: mammalian lethal with Sec13 protein 8
mTOR: mechanistic target of rapamycin
mTORC1: mTOR complex 1
mTORC2: mTOR complex 2
MVB: multivesicular body
NPRL2: nitrogen permease regulator 2-like protein
PI3K: phosphoinositide 3-kinase
PI3KK: PI3K-related kinase
PI(3)P: phosphatidylinositol (3)-phosphate
PLD: phospholipase D
PRAS40: proline-rich Akt substrate of 40 kDa
Raptor: regulatory-associated protein of mammalian target of rapamycin
Rheb: Ras-homolog enriched in brain
Rictor: rapamycin-insensitive companion of mTOR
S6K: ribosomal protein S6 kinase
SESN: sestrin
SH3: Src-homology 3 domain
SH3BP4: SH3-binding protein 4
SLC: solute carrier family
SQSTM1: sequestosome 1
TBC1D7: TRE2-BUB2-CDC16 domain family member 7
TCA: tricarboxylic acid cycle
TFE3: transcription factor E3
TFEB: transcription factor EB
TRAF6: TNF receptor associated factor 6
TSC1: tuberous sclerosis complex 1
TSC2: tuberous sclerosis complex 2
ULK1: unc-51 like autophagy activating kinase 1
ZKSCAN3: zinc finger with KRAB and SCAN domains 3
